# Evolution and hotspots in breast cancer organoid research: insights from a bibliometric and visual knowledge mapping study (2005-2024)

**DOI:** 10.3389/fonc.2025.1604362

**Published:** 2025-09-03

**Authors:** Tao Wu, BaiXin Li, Hao Lei, FuXing Zhao, Zhen Liu

**Affiliations:** ^1^ Breast Disease Diagnosis and Treatment Center, Affiliated Hospital of Qinghai University, Xining, China; ^2^ Department of Urology, Affiliated Hospital of Qinghai University, Xining, China; ^3^ Department of Pathology, Affiliated Hospital of Qinghai University, Xining, China

**Keywords:** breast cancer, organoids, bibliometrics, drug discovery, research hotspots, 3d bioprinting, tumor microenvironment

## Abstract

**Background:**

Breast cancer is the most common malignancy among women globally. Organoid technology has emerged as a pivotal tool in breast cancer research due to its advantages in modeling tumor heterogeneity and the microenvironment. Despite rapid advancements in this field, a systematic bibliometric analysis to delineate research trends and challenges is lacking. This study aimed to analyze the research landscape, hotspots, and future directions in the field of breast cancer organoids from 2005 to 2024.

**Methods:**

Publications related to breast cancer organoids published between January 2005 and March 2024 were retrieved from the Web of Science Core Collection. Bibliometric tools (CiteSpace and VOSviewer) were employed to analyze collaboration networks (countries/institutions), author contributions, keyword co-occurrence clusters, and burst keywords.

**Results:**

Over the past two decades, the annual publication output on breast cancer organoids has shown continuous growth. The 1618 included English publications garnered a total of 7,323 citations, with a mean citation count of 35.20 per article. The United States (n=666) and China (n=257) contributed over 50% of the publications. Harvard University was the most productive institution. Mina J. Bissell authored the highest number of publications (n=17). High-frequency keywords centered on personalized therapy, immunotherapy, and 3D bioprinting. Burst keyword analysis identified “gene expression” and “signaling pathways” as emerging trends (2019–2024). Key research hotspots include the application of patient-derived organoids (PDOs) for drug screening, co-culture modeling of the tumor microenvironment (TME) with immune components, and the integration of 3D bioprinting technologies.

**Conclusion:**

This study represents the first comprehensive bibliometric analysis to elucidate the evolution and research hotspots in breast cancer organoid research in recent years. The findings provide a thorough summary of the major achievements, persistent challenges, and future frontiers within this rapidly advancing field.

## 1 Introduction

As a revolutionary breakthrough in the biomedical field in the past two decades, organoid technology has broken through the traditional construction paradigm of *in vitro* models (1). Based on the lineage plasticity of adult stem cells or pluripotent stem cells, this technology achieves directional differentiation in 3D self-tissue culture system, and finally forms *in vitro* organ bionic system with organ-specific spatial topology and cell heterogeneity (2). Its histological complexity has reached the level of high similarity to the microscopic structure of physiological organs, showing the bionic advantage over the traditional two-dimensional culture system. The two-dimensional single-layer culture model widely used in the past has problems such as structure flatness defects and genetic stability attenuation, especially in the process of primary modeling, it faces bottlenecks such as low patient-derived sample conversion efficiency and rapid cell function degradation (3). In view of these limitations, the researchers developed a three-dimensional tissue engineering culture platform through *in vitro* microenvironment reconstruction technology, allowing cells to reconstruct the intercellular interaction network in a three-dimensional scaffold, so as to accurately reproduce the spatial regulatory mechanisms during organ development. This 3D biomanufacturing strategy not only maintains the long-term stability of tissue homeostasis, but also significantly improves the fidelity of pathophysiological simulations, providing a revolutionary tool for precision medicine research (4). Toshiro Sato et al. (5) successfully cultured mouse intestinal organoids *in vitro*, which opened the prelude to organoid technology. After more than ten years of development, organoids have gradually become a new *in vitro* model for biomedical research and a powerful tool for maintaining the properties of original cells in the near-native state. Over the past decade, 3D cultures of human embryonic stem cells (ES) and induced pluripotent stem cells (iPS) have successfully engineered multiple organ types for culture, These include breast cancer (6), prostate cancer (7), ovarian cancer (8), lung cancer (9), stomach cancer (10), colorectal cancer (11, 12), liver cancer (13), pancreatic cancer (14), and brain cancer (15). For many years, efforts have been made to explore new ways to co-culture immune cells and organoids in various cancers, including breast cancer. Since breast cancer organoids retain their origin characteristics, tumor xenotransplantation and tumor organ models have become key preclinical models for cancer research (16). Breast cancer organoids can be used in immunotherapy. Cai-Ping Sun et al. (17) through the tumor organoid model, can promote preclinical trials of immunotherapy by retaining endogenous matrix components including various immune cells and simulating immunotherapy responses. Breast cancer organoid technology brings great hope for personalized medicine by creating individual tumor organs and implementing high-throughput drug screening. Meanwhile, breast cancer organoid technology provides a solid foundation for personalized medicine by creating individual tumor organs and implementing high-throughput drug screening. Providing the most effective cancer treatments for individual patients (18). In recent years, the application of breast cancer organoid technology in various fields has been growing, and the number of related publications has also increased year by year. Although breast cancer organoids show great potential in preclinical studies, there are still many challenges in this field, such as high cost, long time consuming, and low success rates. Hence, conducting a bibliometric analysis of breast cancer organoid research is essential to comprehensively summarize the existing achievements and delineate the future challenges within this field. In academic research, research performance is usually assessed by bibliometric indicators (number of publications and citations). Bibliometric methods are widely used because of their compact information, ease of processing and objectivity (19). At present, bibliometric analysis is mainly used to analyze the development trend, academic communication and core influence of literature in specific scientific fields. Not only that, bibliometric analysis can also be used to explore leading journals, core author teams, and current research frontiers and trends in the field. Therefore, it is possible to use bibliometric analysis to evaluate the research activities and trends of specific topics and the most prominent research trends in future studies, not only in the literature itself, but also involving authors,keywords, institutions, countries, etc (20). Despite remarkable progress in breast cancer organoids over the past two decades, bibliometric analyses in this field remain very limited. Therefore, this study employed three bibliometric tools (VOSviewer, R-bibliometrix, and CiteSpace) to analyze the overall landscape of breast cancer organoid-related research. It aims to synthesize the achievements attained over the last 20 years, identify key future challenges, and provide researchers with insights into the field while guiding future research hotspots and trends. 

## 2 Materials and methods

### 2.1 Data source and search strategy

As one of the most authoritative and comprehensive database platforms in the world, Web of Science has become the most commonly used database for bibliometric analysis with its rich content and high-quality academic journals. Therefore, this paper uses the Web of Science Core Collection (WoSCC) as the data base. Literature search was performed simultaneously by Tao Wu and Baixin Li using the Science Citation Index Expanded (SCI-EXPANDED) as the citation database. In order to ensure the comprehensiveness and accuracy of the retrieved data, SCIExpanded was selected as the citation index. The search keyword is set to “TS=((“Breast Cancer*” OR “Breast Neoplas*” OR “Breast Tumor*” OR “Mammary Cancer” OR “Mammary Tumor”) AND (Organoid* OR “3D model*” OR “3D culture*” OR “three-dimensional culture*” OR “patient-derived model” OR “primary culture”))AND document type = (ARTICLE OR REVIEW) AND language = (English) “, the time span selected is January 1, 2005 to December 31, 2024. In addition, all valid bibliographic data, including year of publication, title, author name, nationality, affiliation, abstract, keywords, journal name, etc., are stored in the WoSCC database in plain text format. 

### 2.2 Bibliometric analysis and visualization

Bibliometrics is an independent discipline that provides quantitative methods for the review and study of existing literature in a specific field (21). During the analysis, detailed information such as authors, keywords, journals, countries, institutions, references, etc. can be obtained. Visualization helps to reveal the internal connections between this information, such as different authors working on the same topic, the research priorities of different institutions, new theories proposed by existing institutions, and so on. For data analysis and visualization, we use two tools, VOSviewer and CiteSpace, for country and regional co-occurrence, journal dual graph, high-frequency keyword trends, co-citations, literature citations surge, inter-country networks, institutional researchers, and co-occurrence analysis (22, 23).

## 3 Results

### 3.1 Trend of global publications and citations

**Figure 1:**
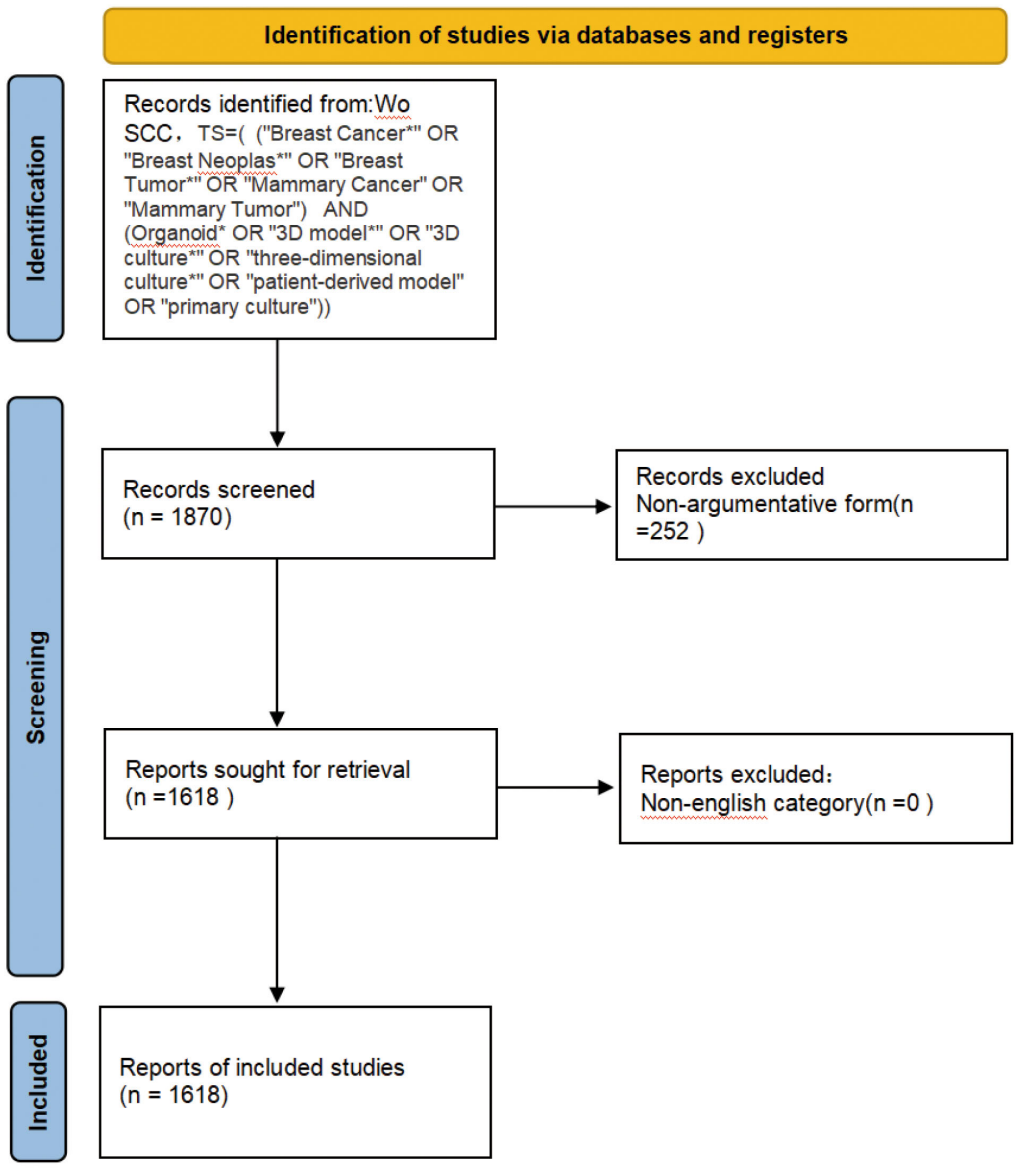
Schematic diagram of the search process.

**Figure 2:**
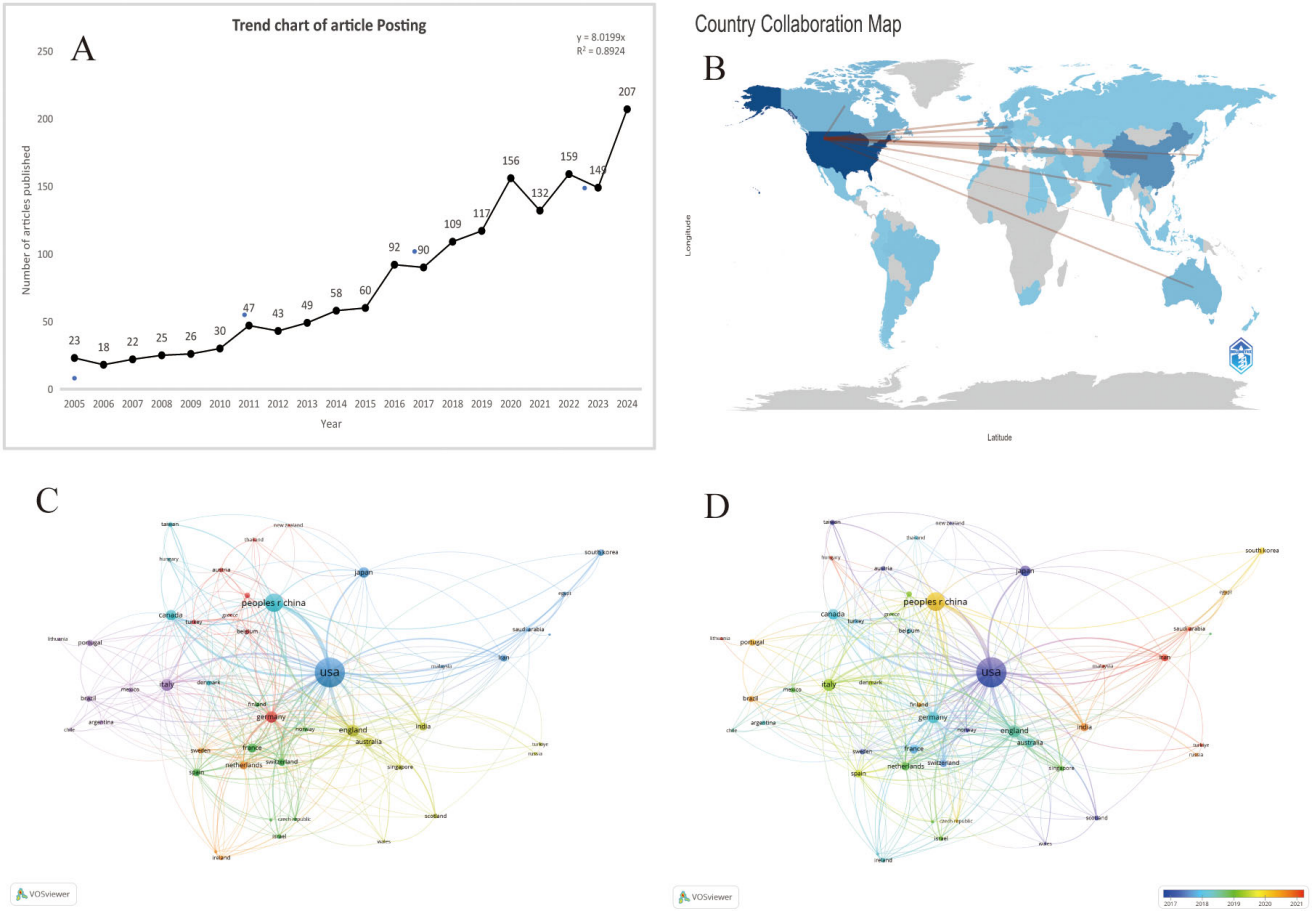
**(A)** Number of publications related to breast cancer organoids by year,2005-2024 . **(B)** Distribution of publications by countriesand regions in the word. **(C)** The citation network of countriesregions mapped through VOSviewer. **(D)** Visualization of citation coerage by country with VOSviewer.

From 2005 to 2024, the Wo SCC collected a total of 1618 articles (**Figure 1**), all of which were published in English. The 1618 papers used in this study, authored by 1,000 authors from 206 institutions in 69 countries and published in 677 journals, represent an increasing number of studieson breast cancer organoids over the last 20 years (**Figure 2A**). These papers received an average of 35.20 citations per paper, for a total of 7,323 citations.

### 3.2 Countries/regions and institutions analysis

According to the World map (**Figure 2B**), publications on this topic come from researchers in 69 countries. The specific number of publications and citations by countries are shown in **Table 1**, among which the United States (666), China (257), India (106), the United Kingdom (105) and Germany (101) contributed the most. In addition, the United Kingdom, Germany and the United States have higher citation rates than China and India. According to **Figure 2C, D**, the citation relationships among 48 countries/regions with at least 5 publications are revealed. Despite publishing more documents, China is second only to the United States in terms of cooperation with other countries.

**Table 1:** Top 10 producing countries related to breast cancer organoids.

Rank	Countries/regions	Documents (N)	Percentage (N/1,618)	Centrality	Citations	Citations per paper
1	United States	666	41.16	0.69	37659	56.55
2	China	257	15.89	0.13	7504	29.2
3	Italy	106	6.55	0.16	2612	24.64
4	England	105	6.49	0.17	3823	36.41
5	Germany	101	6.24	0.11	3899	38.6
6	Canada	82	5.06	0.02	3368	41.07
7	Japan	80	4.94	0.09	3843	48.03
8	Australia	60	3.71	0.04	1992	33.2
9	France	59	3.64	0.15	2590	43.9
10	Netherlands	48	2.97	0.03	1694	35.29

**Figure 3:**
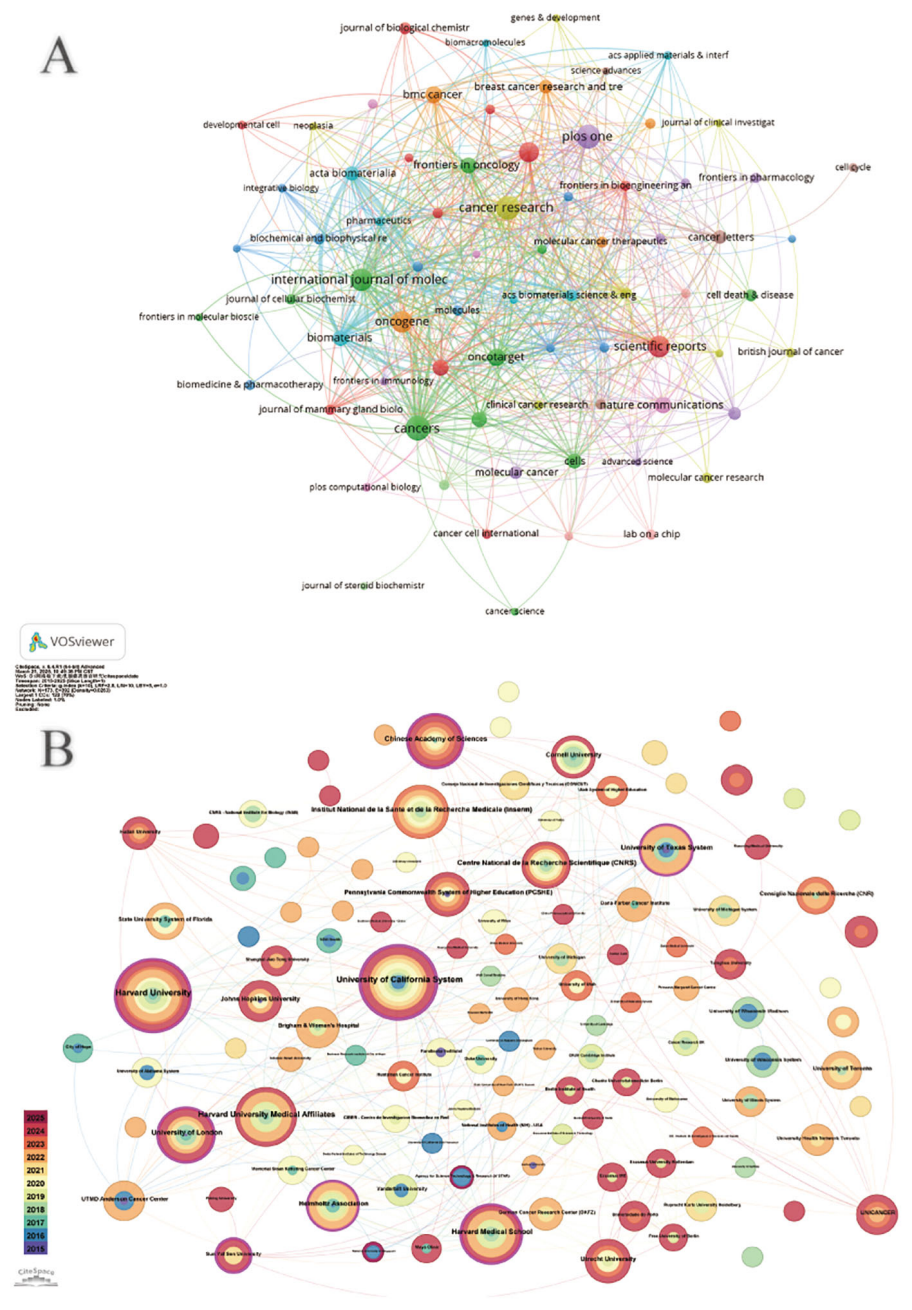
**(A)** Institutional cooperation diagram based on VOSviewer. **(B)** Institutional citation network generated by CiteSpace.

According to CiteSpace, a total of 1,618 papers were provided by 206 different institutions. **Table 2** lists the top 10 institutions with the highest number of published papers. It can be found that the majority of these 10 institutions are from the United States, which is also in line with the country’s literature volume ranking. In **Figure 3A**, a VOSviewer chart of institutional collaboration, American institutions, centered on Harvard University, work more closely together than their Chinese counterparts. In terms of citation analysis of institutions, the top three most cited institutions are University of California, Berkeley (3,886), University of California, San Francisco (3,670), and Harvard University (3,254) (**Figure 3B**). In addition, the chart above shows that work in this area of research started earlier at the University of California, Berkeley. 

**Table 2:** Top 10 institutions ranked by the numbers of publications.

Rank	Institutions	Documents (N)	Citations	TLS	Countries/regions
1	Harvard Med Sch	26	1969	61	United States
2	Univ Toronto	26	1196	43	Canada
3	Univ Calif San Francisco	23	3670	31	United States
4	Univ Texas Md Anderson Canc Ctr	22	1010	24	United States
5	Johns Hopkins Univ	21	1842	10	United States
6	Chinese Acad Sci	20	683	37	China
7	Univ Calif Berkeley	19	3886	23	United States
8	Univ Melbourne	19	799	52	Australia
9	Harvard Univ	18	3254	28	United States
10	Shanghai Jiao Tong Univ	17	507	19	China

### 3.3 Authors and co-cited authors

**Figure 4:**
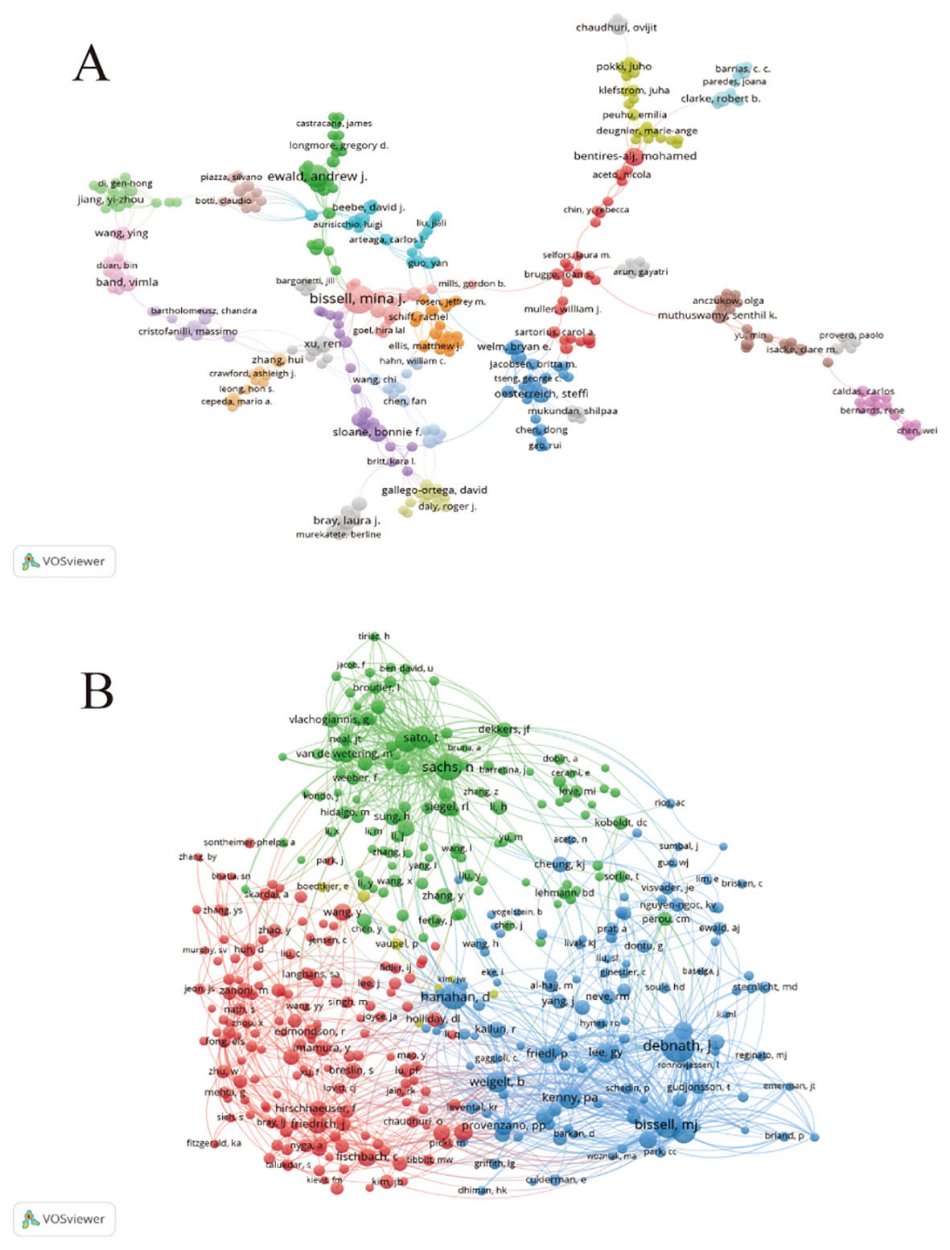
The author cooperation map **(A)** and author co-citation map **(B)** generated by VOSviewer.

An analysis of the authors of publications on breast cancer organoid research gives us insight into the field’s representative scholars and core strengths. **Table 3** lists the top ten authors in the field of organoid research. In terms of number of publications, from Lawrence Berkeley National Laboratory Bissell, Mina J. With the largest number of published papers (17), followed by Ewald, Andrew J (11). Bissell challenged several established paradigms and was a pioneer in breast cancer research. Her work has provided much of the impetus for the current recognition of the important role that extracellular matrix (ECM) signaling and microenvironment play in the regulation of gene expression in normal and malignant cells, and she is also in the top 10 in terms of co-citations, indicating that he has made significant contributions and has a high reputation in the field. In addition to Bissell, Mina J. and Ewald, Andrew J. In addition, the number of papers published by other researchers is basically between 6 and 7, with little difference. Debnath, J., from the University of California, San Francisco, Parnassus Heights, has the most frequently cited paper. Overall, collaboration among authors has been more fragmented, and has been dominated by Bissell, Mina J. As the center. However, there is little collaboration among the top ten authors (**Figure 4A**). In the co-cited author network, the different colored parts reflect the same characteristics of the co-cited author research (**Figure 4B**). Except for the yellow group, which contained fewer researchers, the other three groups were evenly distributed in the number of research directions and were closely related to each other.

**Table 3:** Top 10 authors and co-cited authors in the field of breast cancer organoids.

Rank	Author	Documents	Citations	TLS	Co-cited author	Citations	TLS
1	Bissell, Mina J.	17	3387	38	Debnath, J	279	3242
2	Ewald, Andrew J.	11	1368	16	Bissell, Mj	205	4662
3	Bentires-alj, Mohamed	7	324	14	Sachs, N	196	3678
4	Ibrahim, Toni	7	241	56	Hanahan, D	185	3114
5	Petrikaite, Vilma	7	163	2	Sato, T	153	3179
6	Sloane, Bonnie F.	7	206	18	Weaver, Vm	150	2773
7	Amadori, Dino	6	230	56	Kenny, Pa	141	2934
8	Band, Vimla	6	236	20	Weigelt, B	133	2591
9	Bongiovanni, Alberto	6	230	56	Drost, J	132	2695
10	Bray, Laura J.	6	97	18	Lee, Gy	106	1687

### 3.4 Journal analysis

To better understand the status of publications related to breast cancer organoids, we analyzed the publications of major journals in the field. As shown in **Table 4**, Cancer (impact factor 6.575, Q1) had the largest number of publications (n=46), followed by Cancer Research (impact factor 12.5, Q1), with 43 publications. In addition, 8 journals had impact factors (IF) of more than 1.5, mainly distributed in Q1 and Q2. This indicates that they all have a high academic reputation in the field. Research on breast cancer organoids shows a positive research trend and is more favored by journals with high impact factors. The most frequently cited journal is the American journal Cancer Research (impact factor 12.5, Q1), with a total of 3,671 citations, followed by the American journal Proceedings of the National Academy of Sciences (impact factor 9.4, Q1) and the American journal Cell (impact factor 45.5, Q1). These journals have an important position in the field of organoids.

**Table 4:** The top 10 journals in terms of publication yolume, correlation strength and citation times.

Rank	Journal	Publications	IF	JCR	TLS	Co-cited-journal	Citations	IF	JCR	TLS	Centrality
1	Cancers	46	6.58	Q1	157	Cancer Res	3671	12.5	Q1	282567	O.O8
2	Cancer Research	43	12.5	Q1	97	P Natl Acad Sci Usa	2622	9.4	Q1	209305	0.06
3	Plos One	42	2.9	Q1	79	Cell	2563	45.5	Q1	219457	0.09
4	International Journal Of Molecular Sciences	37	4.9	Q1	99	Nature	2464	40.137	Q1	215160	0.05
5	Oncogene	35	9.88	Q1	55	Plos One	1976	2.9	Q1	170201	0.04
6	Scientific Reports	35	3.8	Q1	50	Biomaterials	1638	12.8	Q1	167319	0.27
7	Rreast Cancer Research	31	6.1	Q1	64	Nat Rev Cancer	1598	72.5	Q1	139766	0.06
8	Oncotarget	24	1.72	Q2	54	Oncogene	1521	9.876	Q1	102664	0.05
9	Bmc Cancer	22	3.34	Q2	48	J Biol Chem	1469	4	Q2	87864	0.02
10	Nature Communications	22	13.8	Q1	42	Sci Rep-Uk	1427	3.8	Q2	143761	0.04

**Figure 5:**
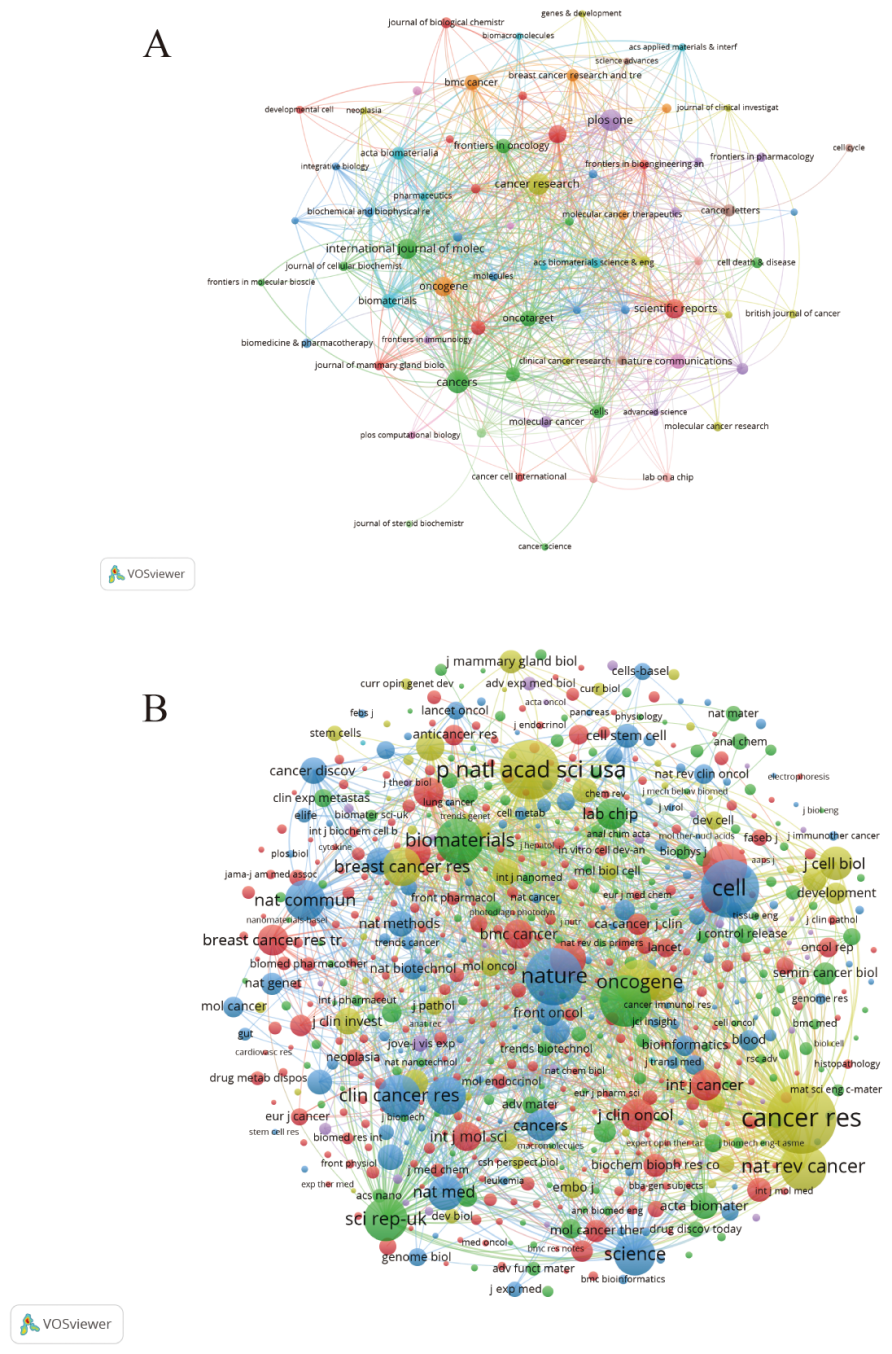
**(A)** Journals related to breast cancer organoids; **(B)** Co-cited journals related to breast cancer organoids.

**Figure 5A** shows the most cited journals, where there is some similarity between journals of the same color. Journals in red include the Journal of Biological Chemistry, Breast Cancer Research and Proceedings of the National Academy of Sciences, which focus on organoid research in breast cancer. Blue journals such as Acta Biomaterials and Biomaterials focus on 3D culture such as Molecular Oncology, Cancer Research and the British Journal of Cancer focus on organoids in immunology. We can observe a strong association between these journals, suggesting that the various directions in organoid research complement and permeate each other. Each circle in (**Figure 5B**) represents a journal, and the size of the circle depends on the strength of the association and the number of citations. Journals with multiple co-citations indicate a strong association between them. For example, Nature, Proceedings of the National Academy of Sciences, and Cancer Research have more common citations and influence. These journals are all related to breast cancer organoid culture raw materials, which indicates that this is the foundation of research in the field of breast cancer organoids. 

### 3.5 Highly cited reference analysis

**Figure 6:**
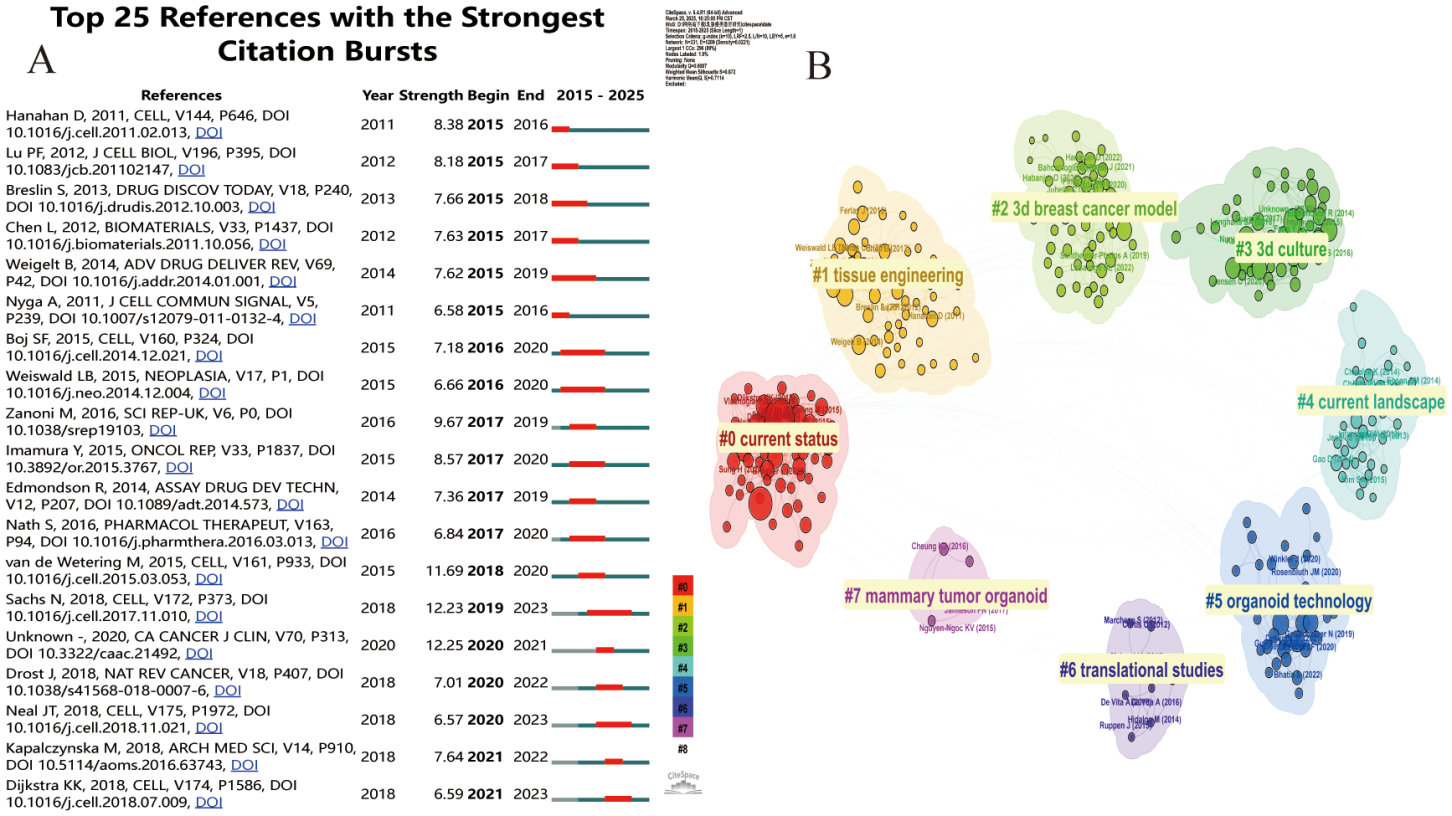
**(A)** Cite Space visualization map of the top 25 most frequently cited breast cancer organoids; **(B)** Cluster view of breast cancer organoids co-cited literature.

By analyzing the top 15 citations (**Table 5**) and the top 25 citations with the highest explosive power in organoid research (**Figure 6A**), we can detect the dynamic change of research direction in this field and provide an indication of future trends. We used CiteSpace software to divide the cited literature into different clusters to better outline the evolution of the topic in the field and the current research status of each research direction (**Figure 6B**). There are a total of eight different clusters. If the number of articles in a cluster is larger, its number is smaller. In this case, the largest cluster is the #0 cluster marked in red, representing the current status of breast cancer organoids, which is closely related to #4, representing the current research status of breast cancer organoids. From the year of publication of the articles represented by each cluster, we can get a rough idea of how organoid research has evolved. 

**Table 5:** Top 15 cited literatures related to breast cancer organoids.

Rank	Author	Article title	Source title	Cited	Year	Category	DOI
1	Sachs,N	A Living Biobank of Breast Cancer Organoids Captures Disease Heterogeneity	CELL	163	2018	Article	10.1016/j.cell.2017.11.010
2	Debnath,J	Morphogenesis and oncogenesis of MCF-10A mammary epithelial acini grown in three-dimensional basement membrane cultures	METHODS	132	2003	Article	10.1016/s1046-2023(03)00032-x
3	Kenny,PA	The morphologies of breast cancer cell lines in three-dimensional assays correlate with their profiles of gene expression	MOL NOCOL	108	2007	Article	10.1016/j.molonc.2007.02.004
4	Lee,GY	Three-dimensional culture models of normal and malignant breast epithelial cells	NAT METHODS	106	2007	Article	10.1038/nmeth1015
5	Hanahan,D	Hallmarks of cancer: the next generation	CELL	104	2011	Review	10.1016/j.cell.2011.02.013
6	Sung,H	Global Cancer Statistics 2020: GLOBOCAN Estimates of Incidence and Mortality Worldwide for 36 Cancers in 185 Countries	CA-A CANCER JOURNAL FOR CLINICIANS	84	2021	Review	10.3322/caac.21660
7	Yamada,KM	Modeling tissue morphogenesis and cancer in 3D	CELL	77	2007	Review	10.1016/j.cell.2007.08.006
8	Drost,J	Organoids in cancer research	NATURE REVIEWS	74	2018	Review	1038/s41568-018-0007-6
9	Van De Wetering,M	Prospective derivation of a living organoid biobank of colorectal cancer patients	CELL	72	2015	Article	10.1016/j.cell.2015.03.053
10	Imamura,Y	Comparison of 2D- and 3D-culture models as drug-testing platforms in breast cancer	ONCOLOGY REPORTS	70	2015	Article	10.3892/or.2015.3767
11	Paszek,Mj	Tensional homeostasis and the malignant phenotype	CANCER CELL	70	2005	Article	10.1016/j.ccr.2005.08.010
12	Petersen,OW	Interaction with basement membrane serves to rapidly distinguish growth and differentiation pattern of normal and malignant human breast epithelial cells	PROCEEDINGS OF THE NATIONAL ACADEMY OF SCIENCES OF THE UNITED STATES OF AMERICA	70	1992	Article	doi 10.1073/pnas.89.19.9064
13	Vlachogiannis, G	Patient-derived organoids model treatment response of metastatic gastrointestinal cancers	SCIENCE	70	2018	Evaluation Study	10.1126/science.aao2774
14	Debnath,J	Modelling glandular epithelial cancers in three-dimensional cultures	NATURE REVIEWS	69	2005	Review	10.1038/nrc1695
15	Sato,T	Single Lgr5 stem cells build crypt-villus structures in vitro without a mesenchymal niche	NATURE	68	2009	Article	10.1038/nature07935

The most influential publications of the past two decades can be divided into early and recent sections. Early studies in this field focused on colony-forming units in #2current status and #19current landscape. During this period, Pengfei Lu et al (24) published that the local microenvironment of cancer cells plays an important role in cancer development. The extracellular matrix provides a theoretical basis for the construction of breast cancer organoids. May help develop new therapeutic interventions by targeting tumor niches. Agata Nyga et al (25) confirmed that 3D *in vitro* models have been used in cancer research, and 3D models can be customized to be bionic and can accurately summarize the natural *in vivo* scenarios in which they are found. These 3D *in vitro* models offer an important alternative, and ways to create more bionic 3D cancer models include, but are not limited to: (i) Provide appropriate matrix components in 3D configurations found *in vivo*, (ii) co-culture cancer cells, endothelial cells, and other associated cells in a spatially correlated manner, (iii) monitor and control hypoxia - mimicking levels found in natural tumors, and (iv) monitor the release of angiogenic factors by cancer cells in response to hypoxia. Building on previous research, in 2014 Britta Weigelt et al (26) used 3D culture models to reveal new pathways in breast cancer and identify accurate biomarkers. Recent high-impact articles relate to disease modeling using breast cancer organoid techniques. Two articles from 2020 cite 3D cultured breast cancer organoids that build better models of tumor response to drugs around cell-cell and cell-extracellular matrix (ECM) in the tumor microenvironment (TME), And it helps cancer researchers willing to use these models to study cancer biology and drug testing (27). And with the advent of human organoids, human organoids offer a unique opportunity to study human diseases and supplement animal models. Through the genetic engineering of human stem cells, human organoids have been used to study infectious diseases, genetic diseases and cancer, and can also be generated directly from patient biopsy samples (28). 

### 3.6 Keyword analysis

In bibliometrics, keyword analysis is one of the key indicators for frontier exploration in the research field. **Table 6** lists the top 20 keywords that appear most frequently. These keywords are all related to each other in some way. Organoid construction techniques rely mainly on two types of core cell resources: adult stem cells (ASSCs) and pluripotent stem cell populations (including embryonic stem cell lines and induced pluripotent stem cell lines). The technology system needs to be coupled with key regulatory molecules, including matrix proteins that maintain the cell microenvironment, FGF family proteins that regulate developmental pathways, Wnt signaling ligands, and bone morphogenetic protein inhibitor Noggin. By integrating a three-dimensional bionic culture system, biomaterials engineering technology, and a microfluidic chip platform, researchers have successfully built a breast cancer organoid model. Such biosimulation systems show important value in basic medical research (analysis of pathological mechanisms), translational medicine applications (development and efficacy evaluation of novel drugs), and clinical practice (formulation of individualized treatment plans and precise tumor diagnosis and treatment). 

**Table 6:** Top 20 keywords with the highest occurrence times.

Rank	keyword	Occurrences	Total link strength	Rank	keyword	Occurrences	Total link strength
1	breast cancer	386	728	11	invasion	35	79
2	3d culture	74	165	12	extracellular matrix	30	93
3	tumor microenvironment	74	191	13	cancer stem cells	28	68
4	organoids	70	163	14	3d models	26	62
5	metastasis	59	147	15	triple-negative breast cancer	26	40
6	cancer	55	125	16	hydrogel	25	87
7	3d cell culture	42	67	17	microenvironment	25	71
8	apoptosis	41	81	18	drug resistance	24	56
9	organoid	37	92	19	tissue engineering	24	86
10	spheroids	36	117	20	drug screening	21	42

**Figure 7:**
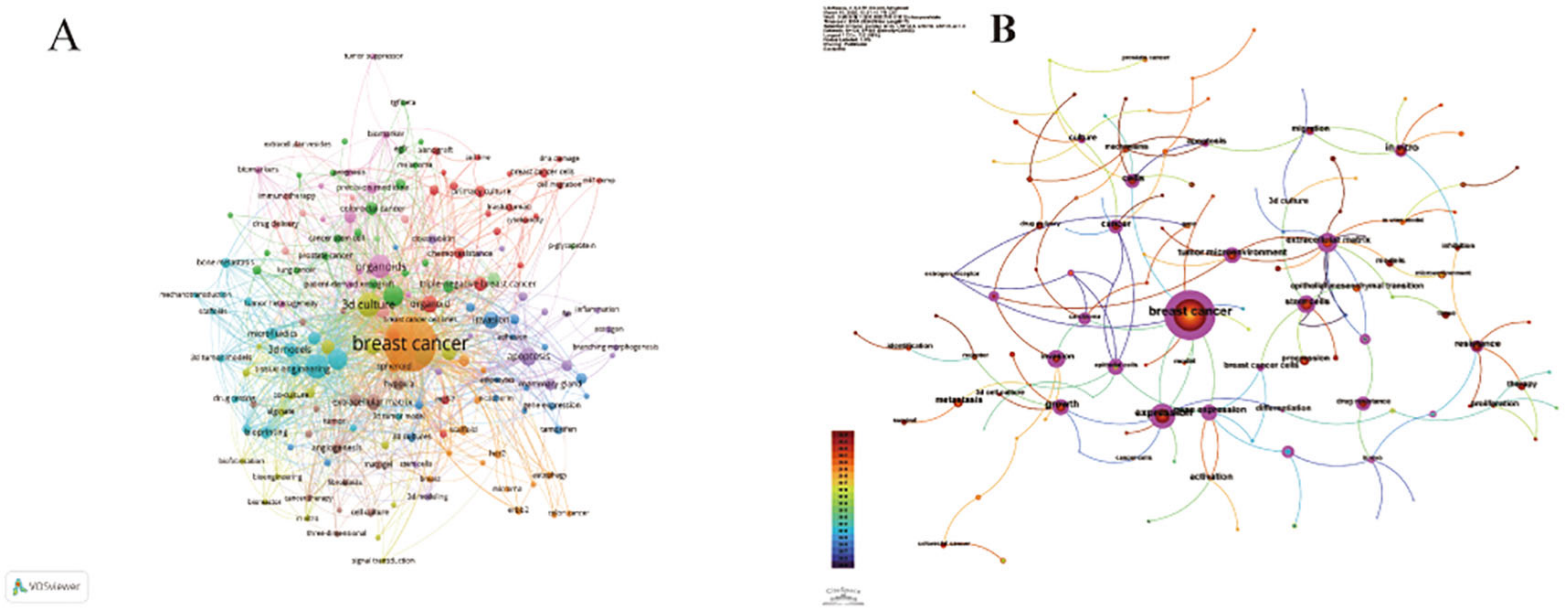
**(A)** Keyword co-occurrence analysis network cluster graph based on VOSviewer. The minimum keyword frequency threshold is 5. **(B)** Keyword contribution graph based on CiteSpace. Each keyword is represented as a node whose size is proportional to the frequency. The lines between the nodes represent the co-occurrence relationship. The distance between nodes indicates the degree of correlation, and the closer the distance, the higher the degree of correlation.

The co-occurrence network diagram and superposition diagram of the author’s keywords can reveal the characteristics of the cluster distribution and time variation of the keywords. According to **Figure 7A**, VOSviewer shows eight major clusters of author keywords. Cyan cluster focuses on “tumor microenvironment”, “three-dimensional model” and “microfluidics”. The pink cluster mainly includes the aspects of personalized treatment and precision tumor diagnosis and treatment. In the purple cluster, apoptosis becomes the focus of research. The blue cluster is mainly related to “invasion” and “migration” related to organoids. The red cluster is mainly around “stem cells” and “biomolecular materials”. In **Figure 7B**, author keywords are displayed in different colors based on the average yearin which they appear. In recent years, terms such as “personalized treatment”, “immunotherapy”, “precision therapy”, “3d biopprinting”, and “stem cell” have frequently appeared, indicating the future research direction and hot spots in the field of breast cancer organoid research. 

**Figure 8:**
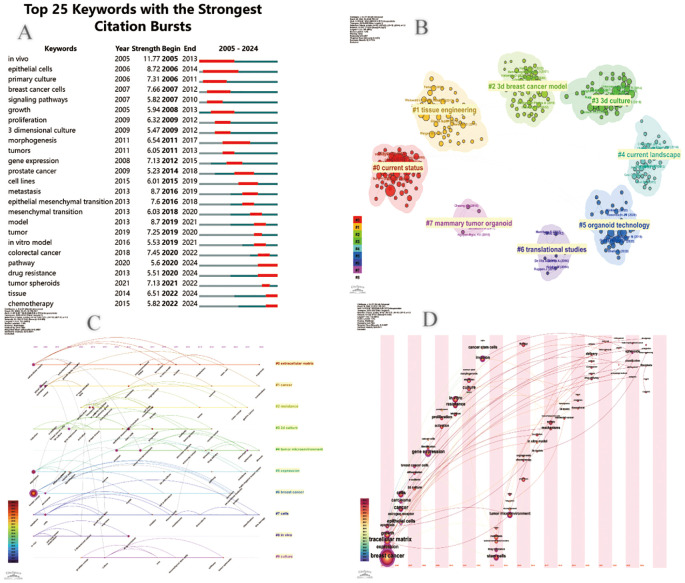
**(A)** Cite Space visualization map of the top 25 most frequently cited breast cancer organoids; **(B)** Cluster view of breast cancer organoids co-cited literature; **(C)** Timcline view of key words of breast cancer organoids; **(D)** Time zone view of breast cancer organoid keywords.

In **Figure 8A**, we also identify research hotspots by keywords with strong reference bursts. The burst of references to keywords like “gene expression,” “*in vitro* modeling,” “3d printing,” and “signaling pathway” continues, suggesting that these terms may translate into new research hotspots. In addition, we use CiteSpace to group keywords into different clusters to get a better overview of how the topic is evolving in the field and how much research is currently being done in each direction (**Figure 8B**). There are 10 different clusters in total. The greater the number of articles that belong to this cluster, the smaller its sequence number. In this case, the largest cluster is the red cluster #0 labeled as the extracellular matrix, which provides the basis for *in vitro* model studies of breast cancer organoids. #1 Cancer and #6 Breast cancer shed light on some of the fundamental research in breast cancer. The early areas of research were mainly reflected in the colony units formed in cells #7 and #8. The breakthrough study by Sato, T (5) published in Nature in 2009, revealed for the first time the unique self-organizing ability of intestinal stem cells labeled by the LGR5 gene. The study, titled “Single Lgr5 stem cells build crypt-villus structures *in vitro* without a mesenchymal niche, “activated the proliferative potential of LGR5-positive stem cells *in vitro*. It was successfully realized that single stem cells independently differentiated into intestinal organoids with crypto-villi polar structure without the support of mesenchymal microenvironment. This milestone achievement not only validates the intrinsic self-organization mechanism of epithelial stem cells, but also creates a paradigm of *in vitro* organoid model construction independent of stromal microenvironment, which lays an important theoretical foundation for subsequent organ regeneration research. As a core technology concept, “3D bioprinting “presents high frequency co-occurrence characteristics with sphere culture system and three-dimensional cell culture technology. By precisely constructing cell assemblies with spatial bionic microenvironments, this technique has become a key methodology for analyzing the mechanism of organ development and realizing the *in vitro* reconstruction of complex tissue structures, and its technical relevance has been particularly prominent in recent organoid research papers. The network cluster diagram (**Figure 8C**) shows the evolution process of each cluster by marking the first occurrence time of each keyword. At present, the most frequently used cluster is #6 breast cancer, while #0 extracellular matrix and #6 breast cancer are among the earliest research directions. Keyword time zone (**Figure 8D**) shows the changes in the research direction and hot spots of breast cancer organoids through the keywords that appear in each year, and reveals the focus of research in the field of breast cancer organoids. 

## 4 Discussion

**Figure 9:**
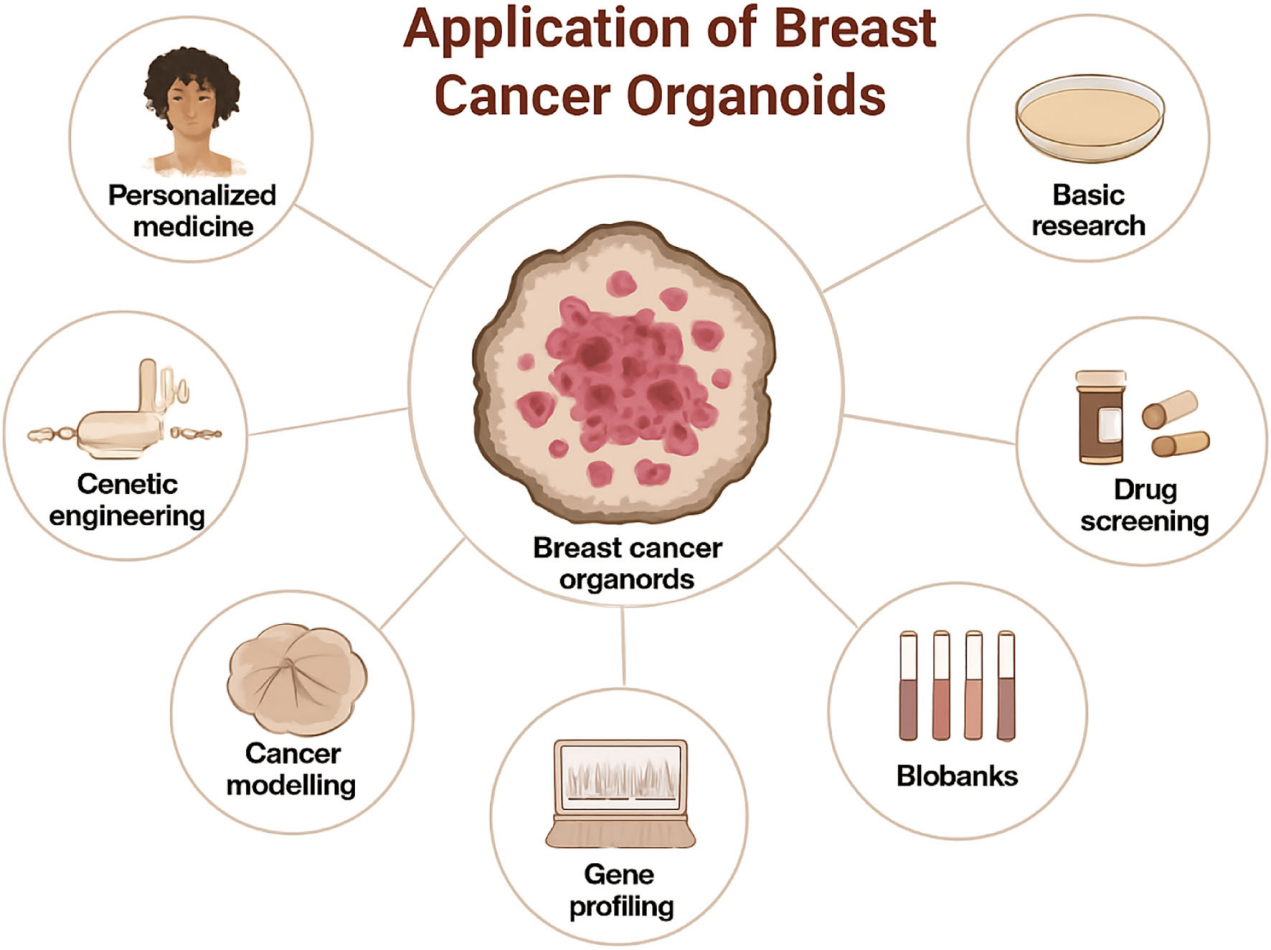
Application of breast cancer organoids.

Keyword analysis helps us identify the current hot research directions in the field of breast cancer organoids (**Figure 9**). These keywords can be divided into four categories: raw materials for organoid culture, organoid culture technology, organoid application, and the type of organoids produced. According to the keyword overlay map, the latter two are the most popular areas of recent research: the application of organoids (drug screening, personalized medicine, precision medicine) and the type of organoids being cultured (patient-derived organoids, 3d printed organoids). These topics are likely to be crucial for future research.

### 4.1 Major achievements in breast cancer organoid research (based on bibliometric evidence)

#### 4.1.1 Successful establishment and application of patient-derived organoids

The application of breast cancer organoids has emerged as a significant research domain. This technology facilitates the testing of diverse anti-cancer therapeutics and guides personalized cancer treatment strategies. By modeling tumorigenesis *in vitro*, these systems enable researchers to observe the entire process of cancer initiation, infection (where applicable), and mutation. Furthermore, organoid technology provides physiologically relevant *in vitro* models capable of elucidating the relationships between infectious agents (e. g. , viruses like HPV or EBV potentially linked to some cancers) and cancer development. Contemporary breast cancer research has historically relied heavily on *in vivo* models and traditional two-dimensional (2D) cell culture techniques. However, these approaches frequently suffer from high failure rates in clinical translation, largely attributable to interspecies differences and the loss of native tissue architecture. To address these limitations, 3D culture systems utilizing organoids—structures that closely recapitulate organ-like morphology and function—have emerged as a highly promising alternative, as pioneered by researchers like Yen-Dun Tony Tzeng et al. (29)Significantly, Ping Chen et al. (30) demonstrated that patient-derived organoids (PDOs) serve as an effective platform for the *in vitro* assessment of patient-specific drug sensitivity, thereby providing critical data to guide personalized treatment decisions for patients with advanced breast cancer. Moreover, Chen Ping et al. (30) highlighted the utility of breast cancer organoids in modeling the complex breast cancer tumor microenvironment (BC-TME). Their research revealed that the BC-TME possesses distinct cellular composition and spatial heterogeneity, factors of profound clinical significance that critically influence therapeutic response.

Researchers have begun exploring the suitability of breast cancer organoids for high-throughput drug screening (HTS) platforms. Typically, these screening procedures aim to simulate clinically applied therapies and are particularly valuable for rare diseases where large-scale clinical trials are unfeasible. The technology enables assessment of the predictive capacity of organoids for drug response by comparing patient outcomes with the responses observed in their matched patient-derived organoids (PDOs).Significant efforts have been dedicated to validating the feasibility of utilizing breast cancer organoids in drug screening applications. For instance, Katrin P. Guillen et al. (31) leveraged matched patient-derived xenografts (PDXs) and PDX-derived organoids (PDxOs) for drug screening. Using a case of early metastatic recurrence in triple-negative breast cancer (TNBC), they demonstrated the feasibility of employing these models for real-time clinical decision-making in precision oncology. This work underscores the substantial potential of breast cancer organoids in predicting drug response.

Breast cancer organoids also enable standardized screening applications. For instance, Zhuolong Zhou et al. (32) employed a high-throughput screening (HTS) platform based on the functional interaction between murine or patient-derived mammary tumor organoids and tumor-specific cytotoxic T cells. This approach facilitated the identification of epigenetic inhibitors that promote antigen presentation and enhance T cell-mediated cytotoxicity. Critically, the standardized measurement of tumor cell-killing activity afforded by this tumor-organoid-T cell co-culture screening system holds significant promise for identifying candidate immunotherapeutic agents across a range of cancers. Furthermore, breast cancer organoids serve as valuable tools for investigating anti-tumor immune responses. For example, Tengku Ibrahim Maulana et al. (33) integrated primary breast cancer organoids to evaluate the efficacy of patient-specific chimeric antigen receptor (CAR) T cells. Their work demonstrates the potential of this platform for broad application in bench-to-bedside translation, thereby accelerating the preclinical development of CAR-T cell products. Similarly, Cai-Ping Sun et al. (17) highlighted the utility of tumor organoid models in facilitating preclinical immunotherapy trials. These models achieve this by either (1): preserving endogenous stromal components, including diverse immune cell populations, or (2) incorporating exogenous immune cells to simulate immunotherapy responses *in vitro*.

Breast cancer organoids serve as powerful platforms for novel drug discovery and identifying patient populations likely to benefit from specific (including existing) therapeutics. For instance, employing breast cancer organoids to validate genomically-driven targeted therapies could significantly aid in selecting personalized drugs for individual patients. Supporting this, Maryam Arshad et al. (34) demonstrated the potential efficacy of neratinib alone or in combination with trastuzumab in HER2-low breast cancer cell and organoid models, providing valuable insights and direction for future research. Additionally, breast cancer organoids are instrumental in exploring potential combination therapies. As exemplified by Sergey V. Nikulin et al. (35), these models were utilized to identify 3,3’-Diindolylmethane (DIM) as a potent microRNA-21 antagonist. This discovery offers novel mechanistic insights and suggests avenues for potentially beneficial chemotherapeutic combinations. Furthermore, breast cancer organoids enable pharmacokinetic (PK) and pharmacodynamic (PD) assessments. In their study, Kristen M. Van Eaton et al. (36) employed organoid models to evaluate hydroxychloroquine (HCQ)-mediated effects on autophagy inhibition, cell proliferation, and cell death, aiming to delineate the role of autophagy in standard anticancer therapies.

Breast cancer organoids also hold potential for applications within nanotechnology. As reviewed by Paz Boix-Montesinos et al. (37), strategies aimed at overcoming limitations in traditional cell culture—including co-culture systems, advanced 3D cell culture, patient-derived cells, bioprinting, and microfluidics (MF)—were explored. Their analysis further proposes the promising development of cancer nanomedicines utilizing breast cancer organoid platforms’ Pranav et al. (38) demonstrated that breast cancer organoids retain the functional physiology of solid tumors, including native gene expression profiles. Critically, the utilization of patient-derived organoids (PDOs) for *in vitro* testing provides crucial support for realizing personalized treatment strategies. Supporting this, Norman Sachs et al. (6) established a well-characterized, representative biobank of breast cancer organoids for cancer research and drug development, coupled with strategies to assess *in vitro* drug responses in a personalized manner. Similarly, Sonam Bhatia et al. (39) highlighted that PDO models recapitulate the intrinsic properties of patient tumors. Specifically, triple-negative breast cancer (TNBC) PDOs serve as robust models for understanding breast cancer biology and progression, thereby paving the way for individualized medicine and tailored therapeutic regimens.

Significant progress has also been made using breast cancer organoids in precision medicine. Cai-Jin Lin et al. (18) revealed, through functional validation in patient-derived organoids, tumor fragments, and *in vivo* models, that precision therapeutic strategies informed by co-occurring alterations hold promise for improving patient outcomes. Furthermore, K Ding et al. (40) leveraged integrative bulk and single-cell analyses of primary tumors and matched PDOs to unveil intra-tumoral heterogeneity, evolutionary trajectories, and actionable therapeutic opportunities for precision medicine. Despite these advances, several limitations persist. Current organoid systems often fail to ideally recapitulate the intricate gene networks and interactions within mature organs, frequently exhibiting immature or “fetal-like” characteristics. This immaturity may constrain their ability to accurately model complex disease phenotypes, particularly those associated with late-onset disorders. Moreover, the long-term culture and maintenance of organoids remain technically complex, especially in the absence of a fully reconstituted native microenvironment, often leading to tissue degradation and loss of function. Additionally, the inherent heterogeneity within and between breast cancer organoid lines can confound data interpretation and potentially impact the reliability of drug screening outcomes.

#### 4.1.2 Advances in modeling tumor microenvironment and immune interactions

Beyond genetic alterations within tumor cells themselves, the microenvironment—composed of diverse non-epithelial cell types, immune cells, and stromal cells—plays a crucial role in tumor growth and eventual metastasis. Conventional tumor models (e.g., 2D cell lines, patient-derived xenografts (PDX)) are severely limited in their ability to dissect the mechanisms of immunotherapy due to their failure to recapitulate the multicellular interactive ecosystem of the tumor microenvironment (TME). Recent breakthroughs in immune-enhanced patient-derived organoids (Immuno-PDOs) have enabled dynamic simulation of the TME by integrating tri-culture systems of tumor cells, stromal cells, and immune cells, providing a revolutionary platform for immunotherapy development. Achieving Functional Reconstruction of Core TME Components. Shijia Liuyang et al. (41) constructed a “TME-on-Chip” microfluidic system modeling tumor-stroma interactions. This system co-cultured patient-derived organoids (PDOs), cancer-associated fibroblasts (CAFs), and endothelial cells within a biomimetic matrix (a collagen/Matrigel hybrid scaffold). Their study demonstrated that CAFs, through the secretion of IL-6 and HGF, activated the EGFR-MAPK signaling pathway in tumors, thereby inducing chemotherapy resistance. Critically, targeting CAFs (e.g., with FAK inhibitors) restored drug sensitivity. This model reproduced, for the first time *in vitro*, the CAF-mediated physical barrier function (via collagen fiber remodeling), elucidating the underlying mechanism of immune exclusion in pancreatic concordance Sun et al. (42) developed an “Immuno-organoid” platform by co-culturing patient-derived organoids (PDOs) with autologous peripheral blood mononuclear cells (PBMCs) or tumor-infiltrating lymphocytes (TILs), supplemented with key cytokines (IL-2, IL-15, IFN-γ) to maintain immune cell viability. Spatiotemporal dynamic monitoring revealed that PD-1 inhibitors promoted the infiltration of CD8+ T cells into the organoid core and facilitated the formation of tertiary lymphoid structures (TLS)—a key histological correlate predictive of response to immunotherapy.

Johanna Englund et al. (43) summarized recent advances in tools and models for breast cancer organoids, covering key aspects such as hormone signaling, tissue architecture, tumor microenvironment (TME), and species-specific mammary gland development, thereby significantly advancing the field. Johanna F. Dekkers et al. (43) employed clustered regularly interspaced short palindromic repeats (CRISPR)-Cas9 to knockout four breast cancer-associated tumor suppressor genes (TP53, PTEN, RB1, and NF1) in mammary progenitor cells derived from six donors. The genetically modified organoids acquired long-term proliferative capacity and, upon transplantation into mice, formed estrogen receptor (ER)-positive luminal tumors. These breast cancer organoid models exhibited responses to endocrine therapy and chemotherapy. Furthermore, using three triple-negative breast cancer (TNBC) lines and four patient-derived xenograft (PDX) models, the study enabled systematic evaluation of tumor latency, growth, and metastasis, offering novel insights for immunotherapy research design and paving the way for patient-specific drug development to ultimately improve clinical outcomes (44). Kelvin K. Tsai et al. (45) leveraged breast cancer organoid models to demonstrate that elevated NCOR2 expression in patient tumors predicts chemotherapy refractoriness, tumor recurrence, and poor prognosis. Their work revealed that targeting the stress- and inflammation-repressing NCOR2-HDAC3 complex could overcome therapy resistance and enhance patient survival. Michael U.J. Oliphant et al. (46) established patient-derived ER+ breast cancer organoid models, providing a robust platform to investigate treatment responses and disease progression in ER+ breast cancer. In 2016, Boussommier-Calleja et al. (47) proposed that microfluidic systems could replicate the complex TME to study tumor-immune interactions. The researchers first analyzed the cellular composition of primary tumors and micro-tumor arrays using flow cytometry.

#### 4.1.3 Integration of 3D bioprinting and advanced engineering technologies

The 3D model in breast cancer organoid technology is the core carrier that simulates the structure, function and pathological features of human organs through an *in vitro* three-dimensional culture system. Its construction breaks through the limitations of traditional 2D cell culture and provides a revolutionary tool for disease research, drug development and regenerative medicine. Although mouse models and two-dimensional (2D) cell culture systems have become research tools in cancer biology, three-dimensional (3D) culture has gained appeal as a new approach that retains the characteristics of *in vivo* biology within *in vitro* systems. Over time, 3D culture systems have evolved from spheres and tumor spheres to organoids, and by doing so, they have become more complex and better representative of primitive tissues. Eleonore Frohlich et al. (48) believe that compared with traditional 2D culture, 3D cancer models can better represent tumor physiology. The important components of 3D models related to physiology were summarized, and the spectra of 3D breast cancer models, such as spheres, organoids, breast cancer on chips and bioprinter tissues, were described. The generation of spheres is relatively standardized and easy to implement. Microfluidic systems allow for the control of the environment and include sensors, which can be combined with spherical or bioprinter models. The intensity of bioprinting depends on the spatial control of cells and the regulation of extracellular matrix. In addition to the main uses of breast cancer cell lines, these models differ in terms of stromal cell composition, matrix and fluid flow.

Recent years have witnessed rapid advancements in 3D *in vitro* models using breast cancer organoids. Anamitra Bhattacharya’s research (49) highlights the critical importance of developing 3D *in vitro* models that recapitulate the complex architecture and physiology of breast tumors. Compared to scaffold-dependent 3D models, scaffold-free *in vitro* disease models better simulate breast cancer pathophysiology by enabling cellular self-assembly/patterning into 3D structures. These models—particularly when incorporating patient-derived primary cells (fibroblasts, endothelial cells, and cancer cells)—demonstrate potential for accurately replicating observed tumor heterogeneity. They also serve as powerful tools for investigating disease molecular mechanisms, identifying novel therapeutic targets, and evaluating treatment modalities. Nakka Sharmila Roy et al. (50) characterized various mesenchymal stem cell (MSC)- and induced pluripotent stem cell (iPSC)-derived 3D breast cancer models that emulate tumor biology, aiming to elucidate potential therapeutic targets. They emphasized that cell- and patient-derived 3D organoid models can advance personalized medicine and accelerate drug discovery pipelines. Natalija Glibetic et al. (51) evaluated state-of-the-art tools for studying cancer metabolism in 3D culture systems, including: Optical Metabolic Imaging (OMI),Matrix-Assisted Laser Desorption/Ionization Mass Spectrometry Imaging (MALDI-MSI),Recent advances in conventional techniques adapted for 3D cultures. Their work further explored progress in identifying and targeting metabolic vulnerabilities for breast cancer therapy. Cristiano Ceron Jayme et al. (52) leveraged 3D organoid cultures to mimic the tumor microenvironment (TME) observed during early human breast cancer progression. This approach enables photo diagnostic strategies for early-stage detection at initial progression phases. It paves the way for on-demand bioprinting of patient-specific organ surrogates, propelling oncology into an era of closed-loop “Design-Validate-Treat” therapeutic paradigms.

The model system for tumor research has gradually matured, with its core lying in more accurately simulating the complexity of human tumors. The successful establishment of patient-derived organoids (PDOs) provides a powerful personalized platform, preserving the key genomic and pathological features of the patient’s tumor, and greatly promoting the exploration of drug sensitivity testing, biomarker discovery, and individualized treatment strategies. Meanwhile, significant progress has been made in modeling the tumor microenvironment (TME) and immune interactions. Researchers are integrating multiple cell types (such as immune cells, fibroblasts, and vascular endothelial cells) and complex biochemical signals to reproduce the dynamic processes of immunosuppression, immune escape, and immunotherapy response in PDOs or more advanced co-culture models. To further enhance the structural mimic ability and controllability of the model, the deep integration of 3D bioprinting with advanced engineering technologies (such as microfluidic chips, intelligent biomaterials, and mechanical force regulation) plays a crucial role, achieving precise construction and manipulation of the TME spatial structure, matrix stiffness, nutrient/drug gradients, and dynamic fluid environment. These three fields - personalized PDOs models, complex TME/immune modeling, and engineered 3D bioprinting platforms - are interwoven and mutually reinforcing, jointly deepening the understanding of tumor biology, accelerating the development and evaluation of novel therapies, and paving the way for more precise and predictive tumor research.

### 4.2 Persistent challenges and future frontiers (identified through bibliometric trends)

#### 4.2.1 Organoid vascularization

Vascularization of tumor organoid models has emerged as a cutting-edge research focus in recent years, with its core challenge lying in the dynamic regulation of neovascularization on drug delivery within the heterogeneous tumor microenvironment (53). Conventional organoid models fail to recapitulate the reduced drug penetration efficiency caused by the tumor vascular barrier due to the absence of a functional vasculature. This results in significant discrepancies between *in vitro* drug sensitivity assays and clinical responses, underscoring the necessity of developing vascularized tumor organoids (VTOs) for precision drug evaluation.

The key bottleneck currently faced in the field of organoid research lies in the limitations of material transport efficiency within the *in vitro* culture system - specifically manifested as the inefficient diffusion of nutrients and the retention of metabolic products. These physical barriers severely restrict the developmental maturity and functional expression of organoids. To address this scientific challenge, the bionic vascular network construction strategy can significantly enhance the efficiency of material exchange and prolong the survival period of organoids by simulating the physiological microcirculation system. The current mainstream vascularization technologies mainly include two research directions: In terms of *in vitro* construction strategies, three-dimensional biomanubicin technology has achieved *in vitro* reconstruction of engineered vascular networks by precisely positioning the spatial arrangement of endothelial cells and other functional cells. This technology preplaces vascular structures in target tissues through a layered printing process, especially demonstrating advantages in the construction of myocardial tissues (54) and Mark vessels (55). In the *in vivo* induction pathway, the microenvironment of the transplanted host can activate the natural angiogenesis mechanism and achieve the self-assembly process of the functional vascular network through the complex cellular interaction between the host and the graft. It is worth noting that microfluidic chip technology, as an emerging platform technology (56), has successfully simulated the three-dimensional microenvironment characteristics of capillary networks *in vitro*, providing an innovative solution for studying the mechanism of angiogenesis and optimizing the organoid culture system.

However, the biomimetic construction of physiological vascular networks faces multiple technical barriers (57):The aberrant branching, disordered spatial topology, and leaky endothelial phenotype characteristic of tumor vasculature require dynamic spatiotemporal control over extracellular matrix-endothelial interfaces at the micron scale. This critically constrains organoid growth dimensions and structural complexity. Effective coupling of capillary beds with tumor parenchyma demands not only structural fusion but also precise coordination of biochemical-biomechanical signaling networks, Gradients of vascular endothelial growth factor (VEGF)Spatiotemporal pericyte recruitment Hemodynamic Force Deficiency: Static culture systems fail to replicate the regulatory effects of hemodynamic shear stress on vascular maturation. This absence of mechanical signaling directly compromises modeling fidelity for critical pathological processes such, Current models cannot adequately simulate tissue-specific drug penetration barriers, leading to inaccurate predictions of drug bioavailability. These collective bottlenecks fundamentally hinder the translational application of vascularized tumor models in key research areas like: Anti-angiogenic therapy development Nanomedicine delivery optimization.

#### 4.2.2 Standardized integration of the complex immune microenvironment

Currently, the organoid field is garnering increasing attention. The advancement of tumor organoid models hinges on reconstructing dynamic interaction networks between malignant cells and the tumor microenvironment (TME). However, conventional approaches face dual barriers: Deficient Immunologic Niches: >90% of tumor organoids contain only epithelial cancer cells, lacking critical immune components such as CD8+ T cells and tumor-associated macrophages (TAMs). This limitation precludes modeling of immune checkpoint blockade responses. Lucie Thorel et al. (58) established the first autologous immune cell-tumor organoid co-culture system, whose clinical implementation holds significant implications for the future of precision oncology. Stromal Biomechanical Distortion: The elastic modulus of Matrigel (2–4 kPa) is substantially lower than that of metastatic foci (8–12 kPa), resulting in attenuated invasive phenotypes. Research by Bauer L. LeSavage demonstrates (59) that engineered matrices combined with patient-derived organoids provide compelling evidence for elucidating how extracellular matrices influence human disease pathophysiology.

Cancer immunotherapies targeting the immune TME show growing validation in clinical trials, yet exhibit dramatically divergent response rates across tumor histologists responses are often transient, idiosyncratic, and confounded by acquired resistance. experimental models capable of recapitulating patient-specific tumor-immune microenvironments—faithfully mirroring tumor biology and immunotherapy effects—would substantially improve: Patient stratification for immuno-oncology Therapeutic target identification and Definition of resistance mechanisms. Such models pave the way for organoid-based immuno-oncology research, drug development, and personalized precision medicine.

#### 4.2.3 Manufacturing cost, throughput and standardization

Currently, as global research in the organoid field deepens, the associated costs are substantial and often underestimated. Key drivers include: 1. Dependence on expensive growth factors in culture media. 2. Increased experimental consumption due to batch-to-batch variability of natural matrices (e.g., Matrigel™). 3. The need for continuous acquisition of patient samples for personalized modeling. 4. The inherent difficulty in scaling production of patient-specific models. Regarding cost control: Traditional organoid culture heavily relies on costly biological reagents and matrices with significant batch variation. Adriana Mulero-Russe et al. (60) reported a promising approach using synthetic polyethylene glycol-laminin (PEG-laminin) hydrogels. This technology not only reduces matrix costs but also minimizes batch variability to <5% through tunable mechanical properties, thereby significantly enhancing the reproducibility of drug screening assays.

Significant throughput enhancement relies on the deep integration of automation and artificial intelligence. Current manual workflows typically process <100 samples per week, and the analysis of high-content imaging data from 3D organoids remains inefficient (61). Carlos A. Tristan et al. (62) established a robotic platform capable of automating all essential steps for hips culture and differentiation under chemically defined conditions. This approach enables the rapid and standardized production of billions of hiPSCs, allowing parallel generation from up to 90 distinct patient- and disease-specific cell lines. Moreover, Alexandra Sockell et al. (63) developed a microwell-based method enabling high-throughput quantitative analysis of image parameters from single-cell-derived organoids. Crucially, these organoids can be subsequently retrieved from their microwells for molecular profiling, facilitating the precise identification of drug-induced subpopulation heterogeneity.

The absence of standardized protocols stems primarily from the lack of unified quality control (QC) systems. Significant variations in passaging procedures and functional assessment methodologies across different laboratories hinder cross-study data comparability. To enable real-time monitoring of culture quality, Johannes Dornhof et al. (64) developed a microfluidic QC chip integrating glucose, lactate, and pH sensors. This system automatically triggers alerts when microenvironmental parameters deviate from set thresholds (e.g., pH > 7.8), thereby preventing functional drift in the organoids. Notably, pioneering progress in standardization has been achieved for high-throughput drug screening (HTS) using breast cancer organoids. Yichun Wang et al. (65) successfully implemented 3D cell culture technology for HTS, demonstrating its utility in drug discovery and development pipelines.

With the implementation of these engineering strategies—establishing standardized protocols, reducing reagent costs, and enhancing both culture success rates and throughput—organoid technology is poised for gradual maturation towards industrial translation. This convergence of advancements enables robust, cost-effective, high-throughput platforms capable of meeting the demands of industrial-scale applications.

#### 4.2.4 Long-term cultivation and maturity

Over the past two decades, organoid technology has advanced rapidly, demonstrating significant potential for modeling human diseases, particularly cancers. However, key challenges remain. A primary limitation in organoid model development is that most organoids fail to fully recapitulate the mature gene networks of adult organs during long-term culture. Instead, they frequently exhibit embryonic or fetal gene expression signatures. For instance, Toshiro Sato and colleagues (5) demonstrated that excessive Wnt pathway activation leads to sustained expression of stem cell markers like LGR5 in intestinal organoids. Lineage tracing experiments further revealed that the Lgr5+ stem cell hierarchy remains largely static within these organoids. This immature state is manifested by high expression of fetal-type transcription factors and aberrant metabolic pathways. Consequently, such organoids are often inadequate for accurately modeling late-onset complex diseases. Similarly, Yuan Guan et al. (66), aiming to investigate the pathogenesis of congenital hepatic fibrosis, designed human liver organoids to study autosomal recessive polycystic kidney disease (ARPKD). These liver organoids persistently expressed fetal markers AFP and DLK1, while lacking the adult hepatocyte marker CYP3A4.Moreover, while the advent of three-dimensional human brain organoids by Mario Yanakiev et al. provides a unique opportunity to study Alzheimer’s disease (AD) in human-relevant model systems, conventional brain organoids lack an aging microenvironment and fail to spontaneously develop β-amyloid (Aβ) plaques.

Furthermore, maintaining long-term organoid functionality faces challenges due to microenvironmental deficiencies. These include inadequate vascularization and lack of biomechanical cues (67), as well as organoid destabilization resulting from cumulative cellular stress (68). Such deficiencies can lead to the disintegration of tissue architecture. For instance, Kouki K. Touhara et al. (69) demonstrated that oxidative damage and telomere shortening cause the loss of the characteristic crypt-villus structure in intestinal organoids, ultimately compromising the reliability of drug response predictions. To address these limitations, researchers are developing innovative strategies:Jingsi Yang et al (70). engineered 3D-bioprinted endothelialized channels within organoids to enhance oxygen/nutrient delivery and improve microenvironmental maintenance. Ricardo Cruz-Acuña et al (71). developed polyethylene glycol-laminin (PEG-laminin) hydrogels with tunable mechanical properties, aiming to enhance the reproducibility of drug screening assays.

Finally, breast cancer organoids often retain the high heterogeneity of the patient’s original tumor, including the coexistence of subclones with distinct molecular profiles (e.g., HER2+/ER+) (6). However, K. Ding, and colleagues (40) demonstrated through scRNA-seq and ATAC-seq profiling of 50 breast cancer organoid lines prior to drug screening that HER2+/ER- subclones can selectively expand during culture. This expansion can lead to false-negative responses to anti-estrogen therapies. Crucially, without validation using single-cell sequencing or spatial transcriptomics, drug screening results may misrepresent the true sensitivity to targeted therapies, potentially overlooking resistant subpopulations. To address this critical challenge of dynamic heterogeneity, Johannes Dornhof et al. (64) developed a microfluidic organ-on-a-chip platform enabling matrix-based heterogeneous 3D culture. This system allows dynamic tracking of differential drug responses by integrating live-cell imaging with single-cell secretome profiling.

### 4.3 Cooperation and data sharing

Future progress in organoid technology will be critically driven by intensified international collaboration. The dominant partnership between the United States and China—which collectively account for ~50% of publications in this field (**Table 1**)—is emerging as a key force propelling breast cancer organoid research. Institutional network analysis (**Figure 3A**) reveals Harvard University as a central hub, demonstrating strong existing collaborations with US institutions (e.g., UC Berkeley, UCSF) and forging nascent partnerships with Chinese centers (e.g., Shanghai Jiao Tong University). However, interactions between institutions from diverse regions remain limited. To overcome translational barriers, we urge the strategic expansion of multinational consortia—particularly among top-producing nations (US, China, Germany, UK, Italy)—to integrate complementary resources, accelerate technical standardization, and validate findings across diverse patient populations.

Concurrently, deep interdisciplinary integration is paramount. Breakthroughs demand synergistic expertise from: 1. Biologists elucidating tumor heterogeneity and signaling pathways; 2. Engineers developing advanced 3D bioprinting, vascularization, and microfluidic systems (addressing Challenges 1–2 outlined in Section 4.2); 3. Clinicians ensuring physiological relevance and clinical applicability of models. Furthermore, prioritizing open science initiatives is essential to transcend research silos. This requires: 1. Establishing living organoid biobanks with standardized annotation of clinical metadata, genomic profiles, and drug response data; 2. Creating FAIR (Findable, Accessible, Interoperable, Reusable) digital platforms for organoid protocols, omics datasets, and phenotypic screens to support meta-analysis and AI-driven discovery; 3. Mandating that funding agencies and journals enforce data/organoid sharing as a publication requirement, mirroring initiatives such as the Human Tumor Atlas Network. Breaking down geographical and disciplinary boundaries and investing in collaborative infrastructure and open data ecosystems will be transformative, propelling the field towards clinically actionable precision oncology.

### 4.4 Limitations

This study has several limitations. First, the investigation relied solely on the Web of Science Core Collection database, excluding other major biomedical databases such as PubMed, Scopus, and EMBASE. Second, the research sample may lack full representativeness due to restrictions in language coverage, publication types, and time frame. Additionally, as databases are continuously updated, some recently published articles may not have been included in our analysis. Consequently, the findings may not fully capture the complete research landscape.

## 5 Conclusions

This bibliometric analysis delineates the dynamic evolution of breast cancer organoid research over the past two decades, highlighting the United States and China as the leading contributors. Key achievements encompass the successful implementation of patient-derived organoids (PDOs) for personalized drug testing and disease modeling, significant progress in recapitulating the tumor microenvironment and immune interactions, and the integration of innovative 3D bioprinting and engineering approaches. Despite these advances, critical challenges persist. These are most notably the integration of functional vasculature, standardization of complex immune co-cultures, and barriers related to cost, scalability, and long-term maturation. This study provides researchers with a comprehensive overview of the field, serving as a valuable resource for: 1. Identifying key stakeholders and potential collaborators; 2. Understanding achieved successes and current bottlenecks; 3. Focusing research efforts on pressing challenges, such as vascularization; 4. Guiding the development of more physiologically relevant and clinically predictive breast cancer organoid models. Addressing these challenges through enhanced collaboration and technological innovation is crucial for realizing the full potential of organoids in advancing breast cancer precision medicine and therapeutic development.

## Data Availability

The original contributions presented in the study are included in the article/supplementary material. Further inquiries can be directed to the corresponding authors.
